# Potent Antibiotic Lemonomycin: A Glimpse of Its Discovery, Origin, and Chemical Synthesis

**DOI:** 10.3390/molecules27134324

**Published:** 2022-07-05

**Authors:** Shunan Tao, Yang Wang, Ran Hong, Sha-Hua Huang

**Affiliations:** 1 School of Chemical and Environmental Engineering, Shanghai Institute of Technology, Shanghai 201419, China; taoshunan@sioc.ac.cn (S.T.); wangyang1@sioc.ac.cn (Y.W.); 2 CAS Key Laboratory of Synthetic Chemistry of Natural Substances, Center for Excellence in Molecular Synthesis, Shanghai Institute of Organic Chemistry, Chinese Academy of Sciences, Shanghai 200032, China; 3 Innovation Research Institute of Traditional Chinese Medicine (IRI), Shanghai University of Traditional Chinese Medicine, Shanghai 201203, China

**Keywords:** aminosugar, antibiotic, biosynthesis, glycosylation, lemonomycin, total synthesis

## Abstract

Lemonomycin (**1**) was first isolated from the fermentation broth of *Streptomyces candidus* in 1964. The complete chemical structure was not elucidated until 2000 with extensive spectroscopic analysis. Lemonomycin is currently known as the only glycosylated tetrahydroisoquinoline antibiotic. Its potent antibacterial activity against *Staphylococcus aureus* and *Bacillus subtilis* and complex architecture make it an ideal target for total synthesis. In this short review, we summarize the research status of lemonomycin for biological activity, biosynthesis, and chemical synthesis. The unique deoxy aminosugar-lemonose was proposed to play a crucial role in biological activity, as shown in other antibiotics, such as arimetamycin A, nocathiacin I, glycothiohexide α, and thiazamycins. Given the self-resistance of the original bacterial host, the integration of biosynthesis and chemical synthesis to pursue efficient synthesis and further derivatization is in high demand for the development of novel antibiotics to combat antibiotic-resistant infections.

## 1. Isolation and Structural Elucidation

**Figure 1:**
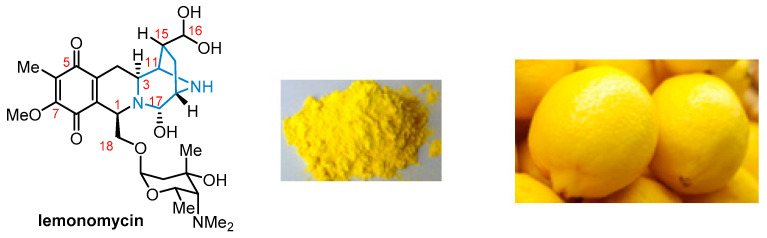
Chemical structure of lemonomycin (**1**), isolated sample, and lemon.

The golden era of antibiotics propelled by the discovery of penicillin has been challenged by a growing crisis in antibiotic resistance worldwide and a decline in investments in the development of novel antibiotics [1]. Natural products are the mainstays of modern antibiotic drugs. Although, only approximately two hundred of 28,000 have been used clinically [2]. The chemical diversity of natural products, including those structures known for decades on the shelf, remains a huge space that has the potential to be a privileged starting point to explore. Lemonomycin (**1**) was first isolated from a fermentation broth of *Streptomyces candidus* (LL-AP191) in 1964 by Whaley and coworkers at the Lederle Laboratory of American Cyanamid Company (Figure 1) [3]. A few grams of lemonomycin hydrochloride were obtained after separation and purification. Its name was based on the color of lemon yellow about the corresponding globular substance. According to the primary ^1^H nuclear magnetic resonance (NMR) and infrared (IR) spectroscopy, the researchers concluded that the structure of lemonomycin should contain hydroquinone, as well as *N*-methyl and *O*-methyl functional groups. At the same time, lemonomycin released dimethylamine after the degradation experiment. The biological evaluation of lemonomycin revealed its broad antimicrobial activity against various microbial pathogens [3,4]. Unfortunately, lemonomycin was found to be fatal at a dose higher than the therapeutic dose, indicating why this potent antibiotic was shelved for decades without further characterization and derivatization.

The comprehensive chemical structure was eventually solved in 2000 by He and coworkers at Wyeth–Ayerst Research (later acquired by Pfizer) [5]. The original sample of lemonomycin hydrochloride (100 mg) [3] was repurified by reversed-phase high performance liquid chromatography (HPLC) to give lemonomycin trifluoroacetate (53 mg). Compared with other alkaloids in the tetrahydroisoquinoline family, the most important feature of lemonomycin is that 2,6-dideoxy-4-amino sugar (lemonose) is connected at the position of C18 through glycosylation [6,7]. Another feature is the existence of a hydrate form of the aldehyde at the position of C16. One of the OH groups of the hydrate can form an intramolecular hydrogen bond, which stabilizes the acetal. The acetal was believed to be the warhead that participates in binding DNA in vivo, as shown in the mode of action of other tetrahydroisoquinolines (THIQs) [6,7]. Lemonomycin and its cyano derivative exhibited strong cytotoxic effects on a human colon cancer cell line (HTC116) in vitro, and their IC_50_ values were 0.36 μg/mL and 0.26 μg/mL, respectively. The minimal inhibitory concentration (MIC) was re-measured to exert 0.4 and 0.2 μg/mL for methicillin-resistant *Staphylococcus aureus* and vancomycin-resistant *Enterococcus faecium*. The absolute stereochemistry of lemonomycin remained elusive at this stage, although the configuration of the aglycon was tentatively assigned according to the absolute stereochemistry of the isoquinoline antibiotics [6,7].

**Figure 2:**
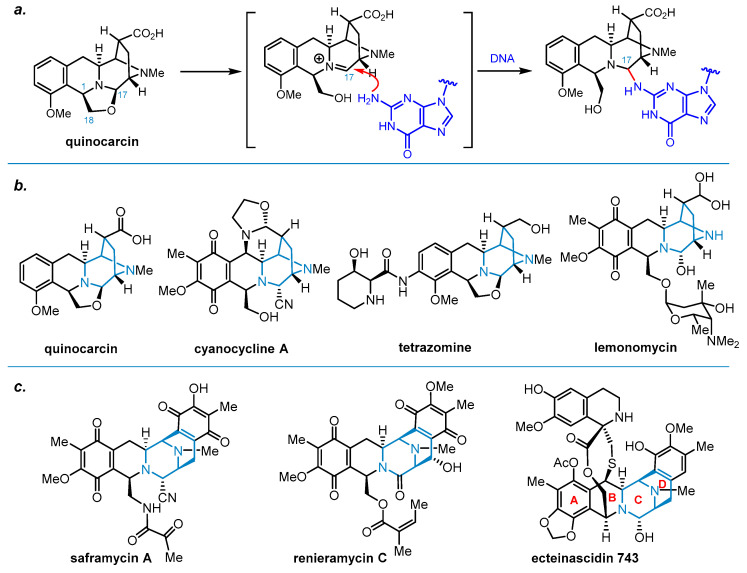
Representative THIQ antitumor antibiotics and mode of action (MoA). (**a**) Mode of action (quinocarcin as example); (**b**) THIQs bearing a diazabicyclo [3.2.1]octane core; (**c**) THIQs bearing a diazabicyclo [3.3.1]nonane core.

Lemonomycin belongs to the family of tetrahydroisoquinoline (THIQ) antibiotics, a family of more than 60 congeners known to have various potent biological activities [6,7]. The antitumor activity arises from the electrophilic iminium ion via a carbinolamine moiety and its equivalents, which directly alkylate the N-2 residue of guanine in the GC-rich region of DNA [6,7,8] (Figure 2a, quinocarcin as an example). THIQ antitumor antibiotics are characterized by the diazabicyclic structure bridged in the tetrahydroisoquinoline ring system. According to the structural features of the bridged diazabicycle, these compounds can be divided into two categories, including two-carbon bridges (e.g., quinocarcin, cyanocycline A, tetrazomine, and lemonomycin) containing a 3,8-diazabicyclo [3.2.1]octane core (Figure 2b) and three-carbon bridges (e.g., saframycin A, renieramycin C, and ecteinascidin 743) containing a diazabicyclo [3.3.1]nonane core (Figure 2c). Given their potent antitumor activity and great potential in the development of antineoplastic drugs, THIQ antibiotics have aroused the enormous interest of synthetic chemists [6,7]. At present, several research groups have completed the synthesis of the THIQ core of lemonomycin, named lemonomycinone amide (**2**, lemonomycin aglycon).

## 2. Current Status of Biosynthesis

**Scheme 1:**
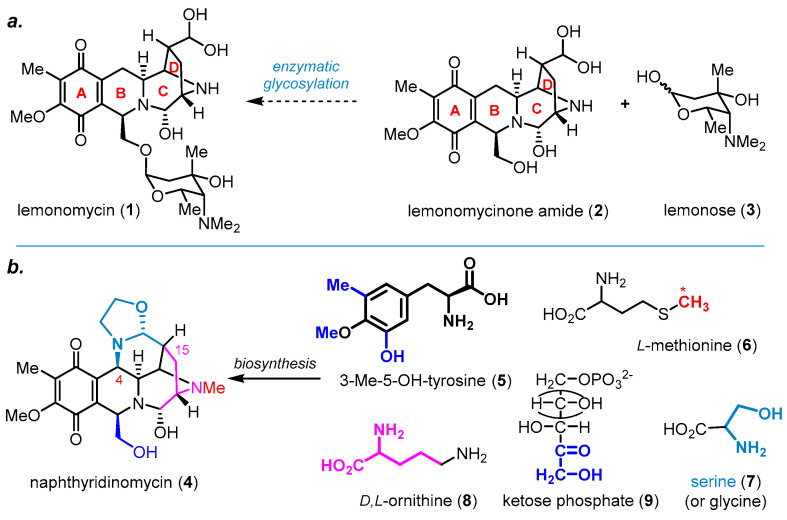
Biosynthetic proposal of lemonomycin (**1**) and naphthyridinomycin (**4**). (**a**) Proposed glcosylation in biosynthesis; (**b**) Biosynthesis of naphthyridinomycin.

The biosynthesis of lemonomycin has not been fully illustrated and literally constructed through the glycosylation of lemonomycinone amide (**2**, aglycon) with lemonose (**3**) (Scheme 1a). Lemonomycin shares a common skeleton with other quinocarcin alkaloids despite the appended deoxy amino acid. Therefore, the biosynthetic pathway can be largely similar to the biosynthetic pathway dedicated to quinocarcins. As the first reported antibiotic in this category, naphthyridinomycin [9,10] (NDM, **4**) has a tetracyclic ring in a nearly identical substituent pattern, except for the C4-amino group and the inverse configuration of the acetal at C15, which constitutes two additional heterocyclic rings in NDM (Scheme 1b). The labeled precursor feeding experiments have established the origin, and the molecular basis from *L*-tyrosine, *L*-methionine, serine (or glycine), *D,L*-ornithine, and ketose phosphates could be incorporated into NDM [11,12,13,14,15,16]. Despite extensive biosynthetic studies of the THIQ core as well as other rare deoxysugars, the biosynthesis of lemonose in vivo and the detailed enzymatic glycosylation remain unclear [17,18].

**Scheme 2:**
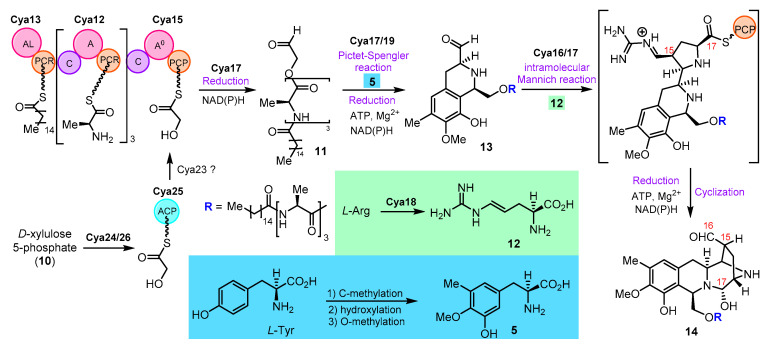
Oikawa’s proposal for the biosynthesis of quinocarcin/SF-1739.

In 2013, Oikawa and coworkers performed gene cluster identification and biochemical analysis of substances in the tetrahydroisoquinoline family, from which the biosynthetic pathways quinocarcin and SF-1739 were proposed (Scheme 2) [19]. On the starting template of Cya13/12, fatty acid and *L*-Ala units were loaded, while the template Cya15 was likely responsible for carrying the glycol unit [14] from the ACP of *D*-xylulose-5-phosphate (**10**) to make its A domain active. Condensation with *L*-aminolevulinic acid and the subsequent reduction delivered aldehyde **11** by Cya17. In the separated pathway, *L*-tyrosine was converted to **5** through a series of *C*-methylation, hydroxylation, and *O*-methylation. By conducting in vitro assays, Cya18 was confirmed to be α-ketoglutarate-dependent dioxygenase catalyzing the dehydrogenation of *L*-Arg to form *L*-(*E*)-4,5-dehydroarginine (**12**). In the Cya17/19 complex, adenylated **5** with Cya19 was transferred to Cya17-PCP, followed by a Cya17-catalyzed Pictet–Spengler reaction with the leader peptide-tethered aldehyde **11** to generate aldehyde **13**, with the assistance of reduction by the Cya17/19 complex. With the activation of **12** under the condition of adenylation provided by Cya16/17, the Cya17-PS domain was used to selectively catalyze the coupling of **12** and **13** via an intramolecular Mannich reaction, reductive cleavage, and spontaneous cyclization to release hemiaminal **14**, bearing an *endo*-configuration at C15, providing an essential carbon framework of SF-1739 and congeners, including aclidinomycins and lemonomycin.

**Scheme 3:**
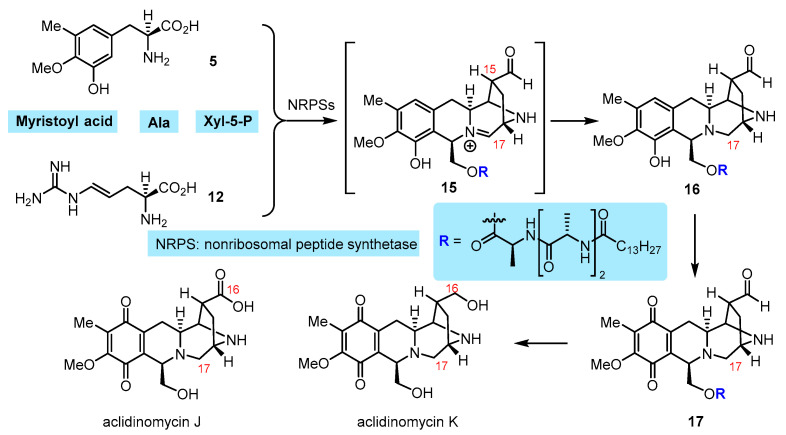
Proposed biosynthetic pathway toward aclidinomycins J and K.

Ju and coworkers recently isolated eleven THIQ-like aclidinomycins from deep-sea-derived *Streptomyces niveus* SCSIO 3406 by genome orientation and the one-strain-many-compounds (OSMAC) strategy [20]. Aclidinomycin J and aclidinomycin K were isolated as amorphous yellow powders and found to have the characteristic core structure of lemonomycin, except for the inverse stereochemistry at C15 and saturation at C17. As indicated in Oikawa’s investigation [19], the specific route is possibly initiated from xylulose-5-phosphate (Xyl-5-P), aminolevulinic acid (Ala), myristoyl acid, a *L*-tyrosine derivative (**5**), and *L*-(*E*)-4,5-dehydroarginine (**12**) as the starting materials (Scheme 3). The discovery of aclindinomycins provides new insights into the molecular logic of the complex polycyclic architecture.

**Scheme 4:**
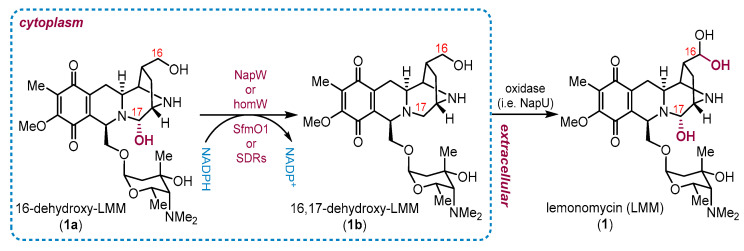
SDR-catalyzed reduction of 16-dehydroxy-LMM (**1a**). (SDR: short-chain dehydrogenase/reductase).

Very recently, Tang and coworkers reported an interesting mechanism of self-resistance when the bacterial host produced lemonomycin and naphthyridinomycin [21]. In the fermentation of *S. candidus* LL-AP191, 16-dehydroxy-LMM (**1a**), was identified instead of LMM (Scheme 4). Large-scale enzymatic reactions were performed to deliver the C17-reduced form **1b**, indicating additional reduction and oxidation steps occurring at different times and locations. As a self-protection strategy, the pharmacophore of the iminium ion at C17 was largely reduced in the cytoplasm to maintain a low concentration for the host. The additional oxidase NapU (flavoprotein) realized the extracellular oxidation step at C17 and C16 to generate the highly potent antibiotic LMM (**1**). The self-resistance strategy poses a challenge to develop a sophisticated biosynthetic pathway to reharvest such potent antibiotics in engineered biosynthetic machinery [22].

## 3. Total Synthesis of Lemonomycin

**Scheme 5:**
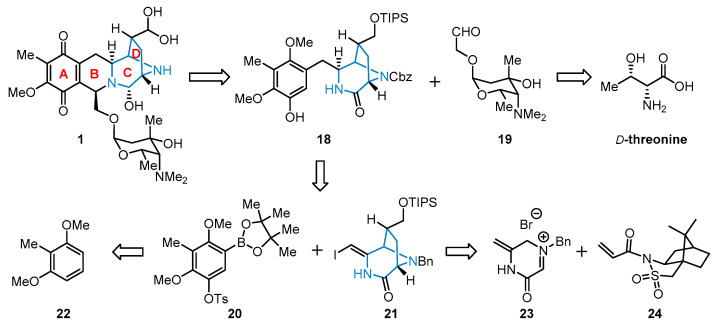
Stoltz’s retrosynthetic analysis of lemonomycin (**1**).

The unique structure and potent antimicrobial activity have attracted considerable interest in several laboratories. Two groups completed the total synthesis, and five groups accessed the aglycon or the key intermediates to constitute the virtual synthesis. The Stoltz group was the first team to disclose the total synthesis of lemonomycin. As shown in Scheme 5 [4,23], the retrosynthetic plan features the Pictet–Spengler reaction of amide **18** with glycosylated aldehyde **19**. Fragment **18** could be readily constructed by the aryl boronic ester **20** and vinyl iodide **21** via Suzuki cross-coupling. The characteristic diazobicyclic intermediate **21** was successfully established between dipolar precursor **23** and Oppolzer sultam derivative **24** by 1,3-dipolar cycloaddition, a novel tactic initially developed by Joule and coworkers [24,25,26] and widely adopted in the syntheses of other THIQ alkaloids [6,7]. Glycosylated aldehyde **19** bearing an amino sugar could be prepared from *D*-threonine in multiple steps.

**Scheme 6:**
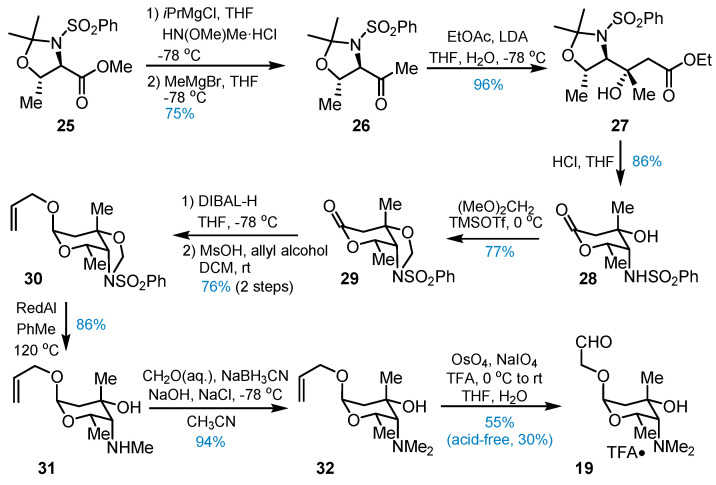
Preparation of aldehyde **19** (the glycoside fragment).

Following the literature-known protocols, methyl ester **25** was prepared from *D*-threonine in three steps by the benzenesulfonyl group replacing the original Cbz protection. After the aminolysis of **25** with *N,O*-dimethylhydroxylamine, the resulting Weinreb amide was converted into methyl ketone **26** in 75% yield after two steps (Scheme 6). Aldol addition with ethyl acetate produced a virtually single diastereomer **27** via the Felkin–Ahn trajectory [27,28]. After treatment of β-hydroxy ester **27** with hydrochloric acid, the corresponding lactone **28** reacted with dimethoxymethane to form oxazolene **29** by using TMSOTf as a catalyst. The subsequent reduction by DIBAL-H and dehydration with allyl alcohol delivered α-allyl ether **30**. The reductive cleavage of the benzenesulfonyl group by Red-Al and the subsequent reductive methylation formed allyl glycoside **32** in excellent yield. The oxidative cleavage of the alkene by Lemieux–Johnson reagent [29] proceeded smoothly to yield aldehyde **19** as a trifluoroacetate salt (55%) and in an acid-free form (30%).

**Scheme 7:**
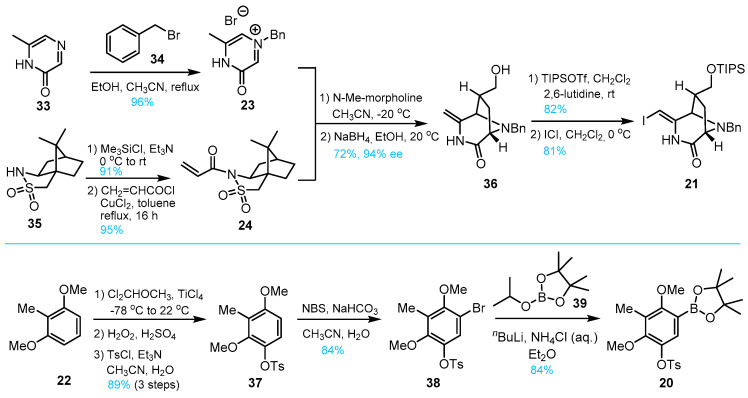
Preparations of diazobicyclic amide **21** and arylboronate **20**.

Bromide salt **23** as a precursor for the requisite 1,3-dipole cycloaddition was readily prepared using benzyl bromide **34** and 6-methyl-2(1*H*)-pyrazinone in anhydrous ethanol and acetonitrile at the reflux in a 96% yield (Scheme 7). However, dipolarophile **24** was obtained by Kocieński’s protocol [30] with silylative activation and the subsequent acylation by acryloyl chloride in the presence of cupric chloride. Accordingly, *N*-Me-morpholine was used for the deprotonation of salt **23** to form an oxidopyrazinium salt for the 1,3-dipolar cycloaddition with **24**. Immediate treatment by NaBH_4_ carried out the reductive removal of the sultam auxiliary to deliver enamide **36** in good yield and a high stereoselectivity (94% ee), indicating an excellent diastereoselectivity achieved in the cycloaddition event. The protection of the primary alcohol with a bulky silyl group and the next iodination produced vinyl iodide **21** as a single isomer in good yield.

**Scheme 8:**
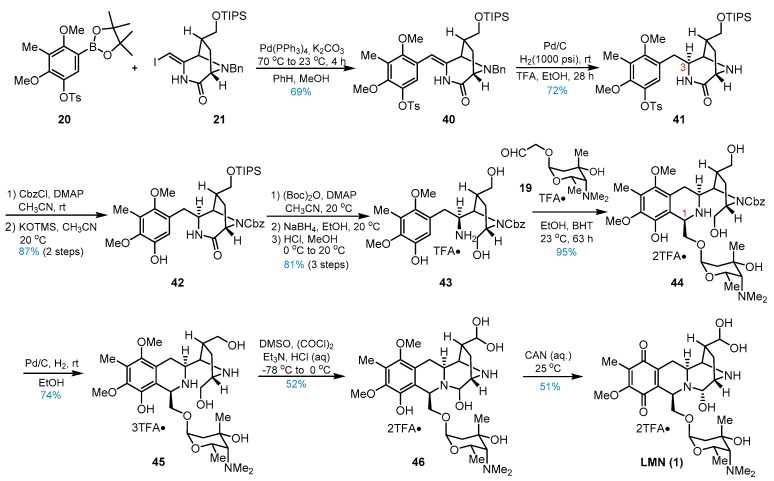
Complete synthesis of (−)-lemonomycin (**1**).

Arylboronate **20** was effectively synthesized from 2,6-dimethoxytoluene **22** in five straightforward steps, including Friedel–Crafts acylation, Baeyer–Villiger oxidation, tosylation, bromination, halogen exchange, and subsequent trapping by the boron ester (Scheme 6). With two fragments **20** and **21** in hand, Suzuki cross-coupling catalyzed by Pd(PPh_3_)_4_ proceeded to deliver the requisite aryl enamide **40** on the gram scale with a good isolated yield (69%) (Scheme 8). The hindered trisubstituted enamine was subjected to heterogeneous hydrogenation at 1000 psi to generate **41** with excellent diastereoselectivity. Reprotection of the amine group by the Cbz group and the subsequent removal of the *p*-toluenesulfonyl group by potassium trimethylsilolate (KOTMS)-promoted hydrolysis released the cyclic amide in **42**. To release amine for the programmed Pictet–Spengler reaction, treatment with (Boc)_2_O enabled the complete reduction of the amide group. The subsequent acid workup produced amino triol **43** as a form of trifluoroacetic acid (TFA) salt in 81% yield after three steps. The key Pictet–Spengler cyclization of **43** with lemonose-derived aldehyde **19** (TFA salt) was performed in a foil-wrapped vial for 63 h to give bis-trifluoronate **44** in a 95% yield. Again, the PS-cyclized product was isolated in the form of a single diastereomer at C1. Hydrogenolysis and Swern oxidation resulted in hemiaminal bis-trifluoronate **46** bearing a hydrate form of aldehyde. Finally, oxidation by cerium ammonium nitrate (CAN) to convert phenol into benzoquinone furnished bis-trifluoronate (−)-lemonomycin (**1**), which was completely identical to the reported data [5], and thus fully validated the stereochemistry of lemonomycin.

**Scheme 9:**
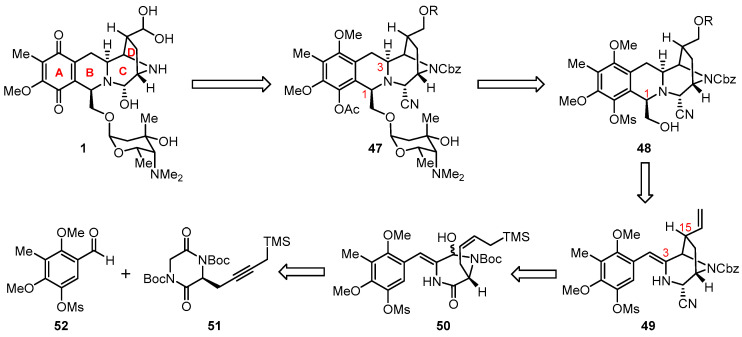
Fukuyama’s retrosynthetic analysis of lemonomycin.

Given novel aminosugars in various potent antibiotics, the Fukuyama group proposed a late stage of glycosylation in the synthetic design to be versatile for future derivatization. In 2012, the total synthesis of lemonomycin was reported by this group [31]. As shown in Scheme 9, aglycon **48** was anticipated by reducing the enamine in **49** and its subsequent cyclization via the Pictet–Spengler reaction. The 3,8-diazabicyclo [3.2.1]octane skeleton was established by the Hosomi–Sakurai reaction, a strategy being executed in their previous syntheses toward quinocarcin and other alkaloids [32,33,34]. Critical intermediate **50** could be generated by a Perkin-type reaction of diketopiperazine **51** and aldehyde **52**. The latter aldehyde was derived from 2,6-dimethoxybenzene in five steps by a modification of Stoltz’s route [22].

**Scheme 10:**
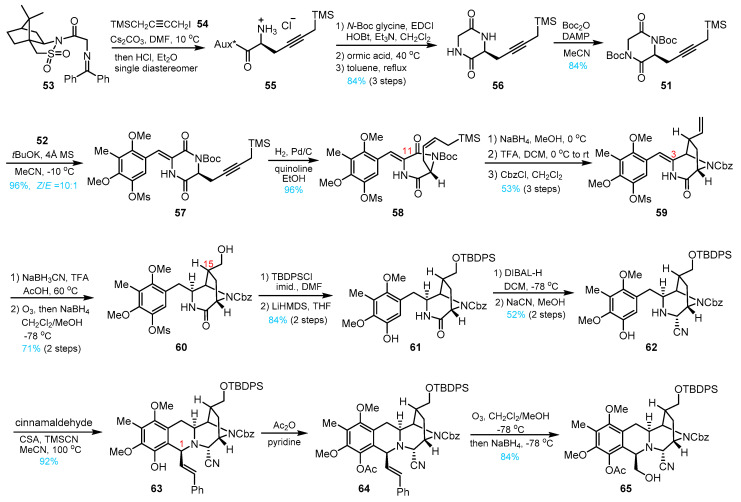
Fukuyama’s synthesis of lemonomycin—preparation of aglycon **65**.

The synthesis commenced with the application of glycine-derived Oppolzer sultam **53** for asymmetric alkylation with propargylic iodide **54** [35] to deliver a single diastereomer of **55** after hydrolysis in an ether solution of HCl (Scheme 10). After condensation with *N*-tert-butyloxycarbonyl (Boc) glycine, the intermediate derived from the acidic removal of the Boc group was then cyclized to release diketopiperazine **56** in a combined 84% yield after three steps. Two secondary amides were masked by the Boc protection, and the resulting cyclic amide underwent Perkin-type condensation [36], with aryl aldehyde **52** to favor (*Z*)-enamine **57** (*Z*/*E* = 10:1) in an excellent yield. The triple bond in **57** was partially reduced by the Lindlar catalyst, while the carbonyl group at C11 was reduced to hemiaminal with NaBH_4_ in an aqueous methanol solution. The acid-promoted Hosomi–Sakurai reaction successfully constructed the bridged ring of **59** with almost perfect stereoselectivity. Reprotection of the amine with the Cbz group gave lactam **59** in a 51% overall yield in three steps. In contrast to Stoltz’s synthesis, trisubstituted enamine was smoothly reduced by NaBH_3_CN under acidic conditions, and the stereochemistry at C1 was satisfied. Ozonolysis and immediate reduction furnished a hydroxymethyl group at C15 in **60**. Functional group manipulations, including protection by the tert-butyldiphenylsilyl (TBDPS) group and deprotection of the mesyl group by lithium bis(trimethylsilyl)amide (LiHMDS) [37], generated the phenol derivative **61**. To improve the reactivity of the amine, the lactam group was first reduced by diisobutylaluminum hydride (DIBAL-H), and the resulting hemiaminal was converted to amino nitrile **62**. To complete the synthesis of the aglycon, cinnamaldehyde was merged into the desired Pictet–Spengler cyclization in the presence of CSA and TMSCN, and then the tetracyclic skeleton **63** was formed at 92%. The high stereoselectivity achieved was found to be crucial under thermodynamic control. After acetylation, the requisite aglycon **65** was completed in an 84% yield after ozonolysis and subsequent reduction.

**Scheme 11:**
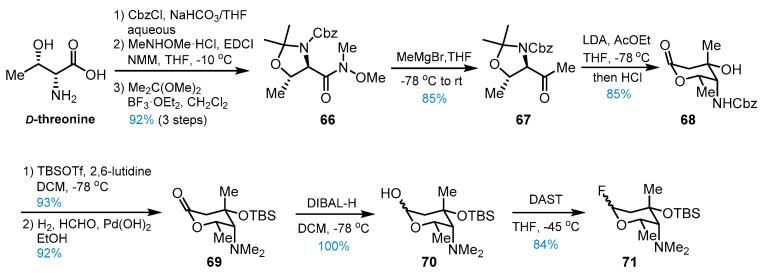
Fukuyama’s synthesis of (−)-lemonomycin—preparation of glycosyl 71.

The synthetic route of lemonose **71** was slightly modified from the previous procedure [22]. The initial amine protection group was chosen as the Cbz group instead of the benzenesulfonyl group in a previous synthesis (Scheme 11). After the formation of methyl ketone **67** through Weinreb amide, the aldol reaction and deprotection afforded lactone **68**. The tertiary alcohol was protected by the tert-butyl(dimethylsilyl (TBS) group, followed by the removal of the Cbz group and reductive amination in a one-pot method. The corresponding amine **69** was subjected to partial reduction by DIBAL-H to release acetol **70**. Finally, fluorination by treatment with dimethylaminosulfur trifluoride (DAST) [38] resulted in glycosyl fluoride **71** in 84% yield.

**Scheme 12:**
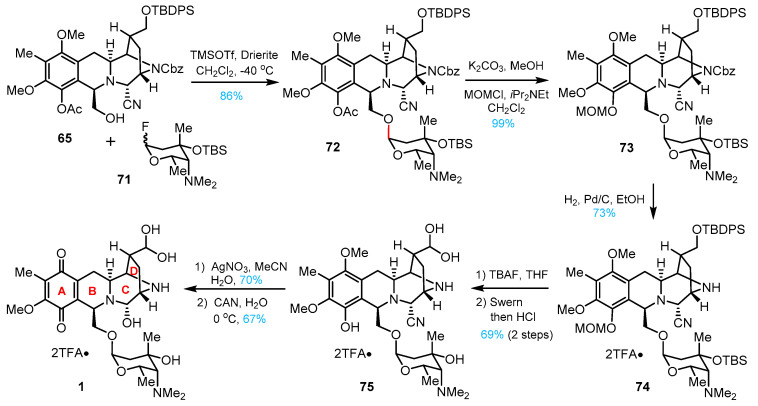
Fukuyama’s synthesis of (−)-lemonomycin (**1**).

With aglycon **65** and glycosyl fluoride **71** in hand, glycosylation was promoted by trimethylsilyl trifluoromethylsulfonate (TMSOTf) to give α-anomer **72** in 86% yield (Scheme 12). After the protection exchange, the fully protected compound **73** was subjected to debenzylation by hydrogenolysis and the subsequent removal of two silyl groups by tetra-*n*-butylammonium fluoride (TBAF) to generate amino alcohol **74** as a bistrifluoronate salt. Subsequent Swern oxidation smoothly converted the primary alcohol into hydronated aldehyde **75** after acidic workup. Silver nitrate was then adapted to remove the CN group to rebound the hemiaminal functionality, which was also tolerant to the final oxidation by CAN to complete the total synthesis of lemonomycin (**1**). This strategy diminished the separation steps of polar intermediates, which are clearly tedious and troublesome to handle because of the given salt forms in the late stage of total synthesis.

## 4. Other Studies toward Lemonomycin

**Scheme 13:**
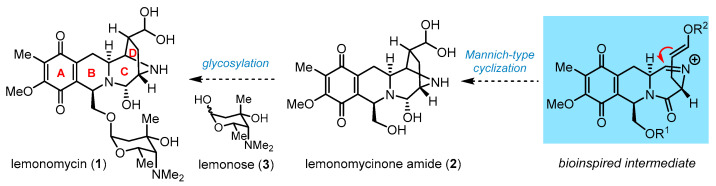
A general bioinspired approach of Magnus and Zhu’s syntheses.

The synthetic works contributed by the Magnus and Zhu groups were essentially inspired by the late stage of the biogenesis of quinocarcin alkaloids (Scheme 2). They designed the pathway, including the construction of lemonomycinone amide (**2**, aglycon) via a Mannich-type cyclization, to furnish the bridged ring system and final glycosylation with lemonose (Scheme 13).

**Scheme 14:**
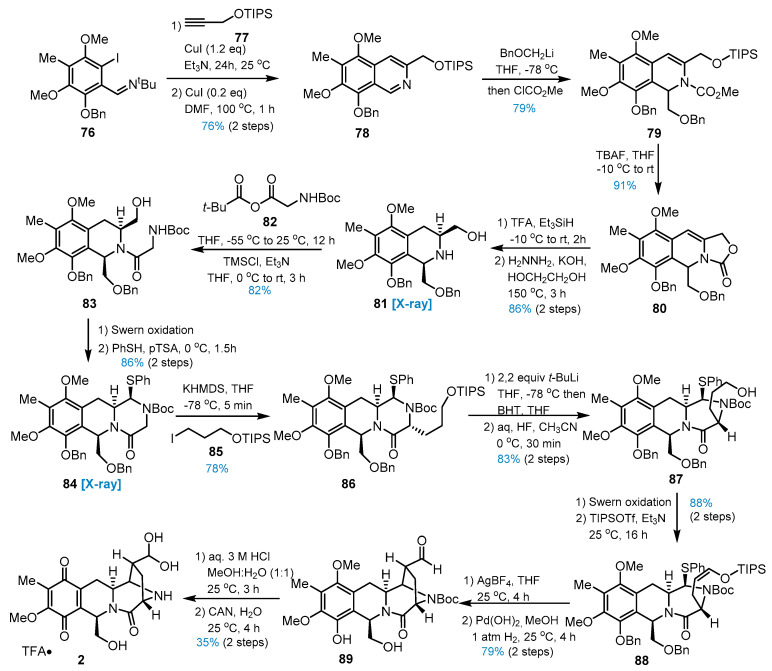
Magnus’ synthesis of lemonomycinone amide (**2**).

In 2005, Magnus and coworkers devised a Mannich reaction-based strategy to construct the aglycon, lemonomycinone amide (**2**) [39,40]. The synthesis began with 2,4-dimethoxy-3-methylbenzaldehyde as the starting material to prepare a fully substituted aryl aldehyde-derived imine **76** in a seven-step sequence [41]. Copper-mediated cross-coupling between **76** and silylated propargyl alcohol **77** under Castro conditions [42] and subsequent aromatization resulted in isoquinoline **78** in a 76% overall yield (Scheme 14). The subsequent addition of benzyloxymethyllithium [43] and methyl chloroformate furnished the substitution on the B-ring. The removal of the triisopropylsilane (TIPS) group by TBAF also promoted cyclization to generate oxazolidinone, which was suitable for ionic hydrogenation by TFA-Et_3_SiH and immediate hydrolysis to afford *cis*-amino alcohol **81** in a high yield. After structural confirmation by X-ray analysis, compound **82** was introduced into a glycine motif via an activated reagent, *n*-tert-butoxycarbonyl glycine. Swern oxidation initiated the formation of hemiaminal in a mixture of diastereomers (*dr* 3/2) [44], which was further converted into the thermodynamically stable thioaminal **84** (X-ray) in excellent yield. Single diastereomeric **86** was obtained from the alkylation of **84** and iodide **85** by potassium bis(trimethylsilyl)amide (KHMDS) as a base. To invert the stereochemistry at C13, the treatment of **86** by *^t^*BuLi and trapping by a hindered proton source, butylated hydroxytoluene (BHT), gave the α-configured isomer which was desilylated to produce alcohol **87**. After Swern oxidation, the resulting aldehyde was converted to silyl enolate **88** in 88% yield. The key Mannich-type cyclization was initiated by thiophile AgBF_4_, and a single stereoisomer at C15 of the aldehyde was obtained. The high stereoselectivity is largely attributed to the steric bulk of the C1 substituent (BnOCH_2_) in the axial orientation. After hydrogenation of two benzyloxy groups by Pearlman’s catalyst Pd(OH)_2_ [45], alcohol **89** was subjected to the removal of the Boc group and hydration of the aldehyde group at C15 under acidic conditions. The final oxidation by CAN completed the synthesis of lemonomycinone amide (**2**) in 35% yield as a racemic form.

**Scheme 15:**
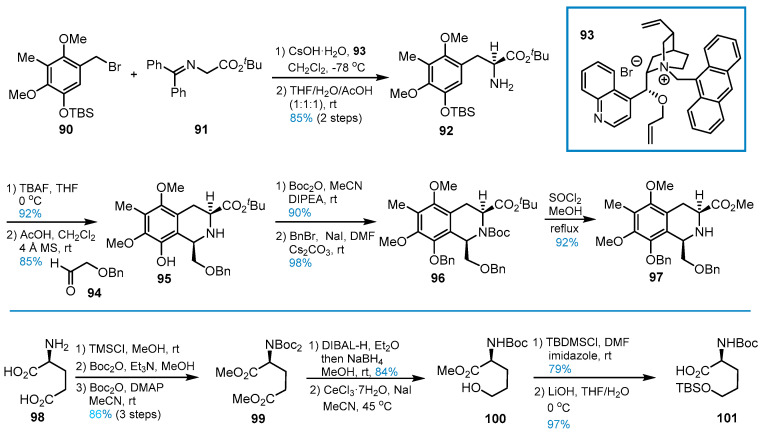
Zhu’s synthesis of lemonomycinone amide—preparations of **97** and **101**.

In 2008, the Zhu group devised a similar Mannich-type strategy to synthesize lemonomycinone amide (**2**) in an optical form [46]. In contrast to the abovementioned strategies, they first established the C13 stereochemistry by the asymmetric alkylation of glycine-derived imine **91** with functionalized benzyl bromide **90** in the presence of the Corey–Lygo phase transfer catalyst **93**, [47,48,49] O-(9)-allyl-*N*-(9′-anthracenylmethyl) cinchonidium bromide, producing amino ester **92** in an 85% yield (Scheme 15). The subsequent addition of TBAF for desilylation to release the phenol group enabled the Pictet–Spengler reaction of benzyloxy acetaldehyde **94** to deliver a single cyclized product **95**. After protection and ester exchange, compound **97** was obtained in excellent yield. To circumvent the linear manipulation in the Magnus synthesis [40], the Zhu group adopted *L*-glutamic acid (**98**) to introduce the four-carbon unit. However, the separated synthesis of **101** required seven steps to install the proper functional groups. After three steps of protection, compound **99** was subjected to reduction [50] at the distal ester. The subsequent additional three steps of protection and deprotection gave amino acid **101**.

**Scheme 16:**
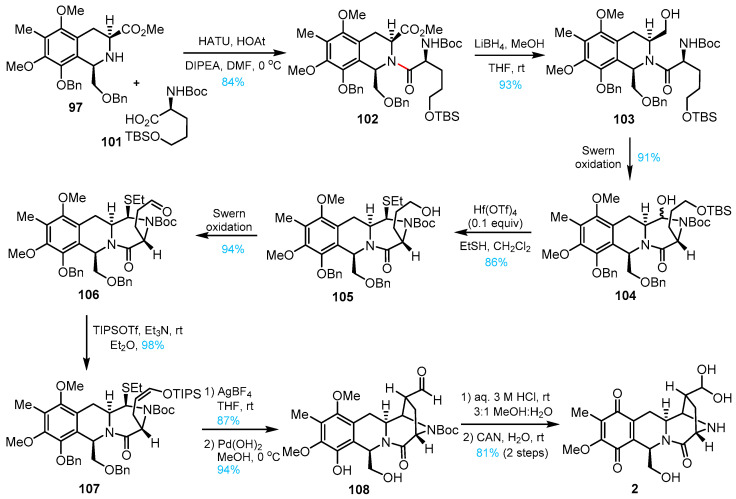
Zhu’s synthesis of lemonomycinone amide (**2**).

The condensation of isoquinoline **97** and amino acid **101** was carried out to produce amide **102** without detectable epimerization in the presence of hexafluorophosphate azabenzotriazole tetramethyl uranium (HATU) and N,N-diisopropylethylamine (DIPEA) in dtmethylformamide (DMF) with a yield of 84% (Scheme 16). The C-ring was formed by Swern oxidation after **102** was reduced to alcohol **103** by LiBH_4_ to form semiacetal **104** as a mixture of diastereomers (*dr* 3/2). Subsequent treatment with Hf(OTf)_4_ [51] and EtSH delivered alcohol **105** as a single diastereomer under thermodynamic control. After Swern oxidation and the subsequent formation of silyl ether by TIPSOTf and triethylamine, the programmed Mannich-type cyclization promoted by AgBF_4_ proceeded smoothly to deliver the requisite aldehyde with the correct stereochemistry. Following Magnus’s condition [40], debenzylation by Pd(OH)_2_ afforded alcohol **108**. After the modification of the previous hydrolysis under acidic conditions, the aldehyde group was hydrated and then oxidized by CAN to give lemonomycinone amide (**2**) in an 81% yield.

**Scheme 17:**
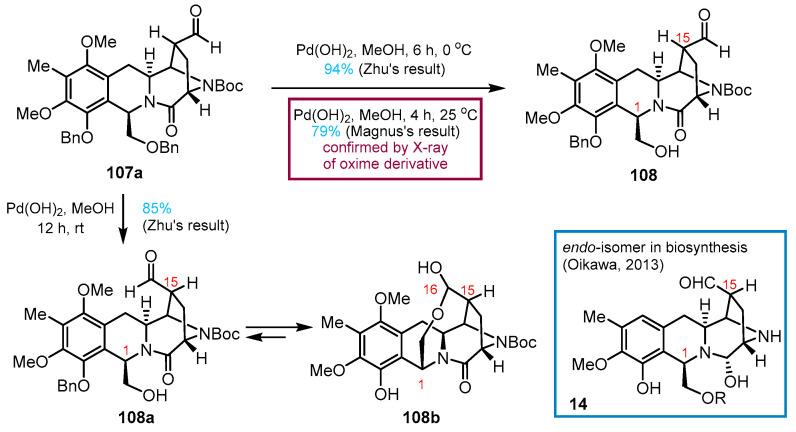
A facile epimerization at C15 for biosynthetic implication.

A particularly interesting phenomenon was discovered during the hydrogenolysis of **107a** by the Perlman catalyst (Pd(OH)_2_) [45] to remove two benzyl groups at room temperature (12 h at r.t. versus 6 h at 0 °C). In contrast to Magnus’s observation [39,40], the *endo*-isomer **108a** (15-*epi*-**108**) was isolated in an 85% yield under identical conditions (Scheme 17). Although the *endo*-isomer is thermodynamically less favorable for steric reasons, the proximity of the C1-hydroxymethyl group may drive the equilibrium toward the final formation of hemiacetal **108b**, providing mechanistic insights into the stereochemical outcome of the kinetically favored *endo*-adducts in the proposed biosynthesis [19].

**Scheme 18:**
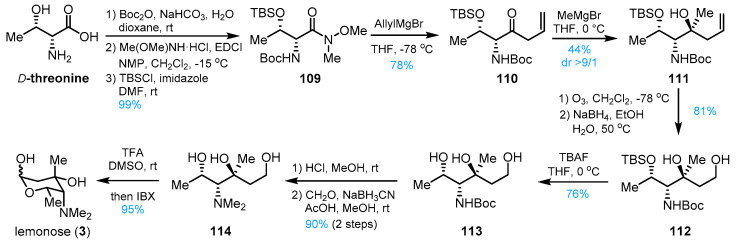
Zhu’s synthesis of lemonose (**3**).

Three years later, Zhu and coworkers continued to finish the asymmetric synthesis of lemonose (Scheme 18). [52] Threonine was again selected as the chiral pool, which was first converted to Weinreb amide **109** after three steps. Subsequently, two additions by different Grignard reactions readily furnished **111** with all requisite stereogenetic centers. The terminal double bond was then subjected to ozonolysis and a subsequent reduction to deliver alcohol **112** in an 81% yield for two steps. After global deprotection, double reductive amination with HCHO in the presence of NaBH_3_CN furnished **114**. To attenuate the impact of tertiary amines, the pretreatment of trifluoroacetic acid successfully chemoselectively converted the primary alcohol to aldehyde by *O*-iodoxybenzoic acid (IBX) [53] and then cyclized to form lactol **3** (lemonose) in a total of 10 steps and an 18% overall yield. However, the complete assembly of lemonmycin (**1**) was not disclosed despite that both the given glycosylation of aglycon **2** with protection-free lemonose (**3**) were obtained.

**Scheme 19:**
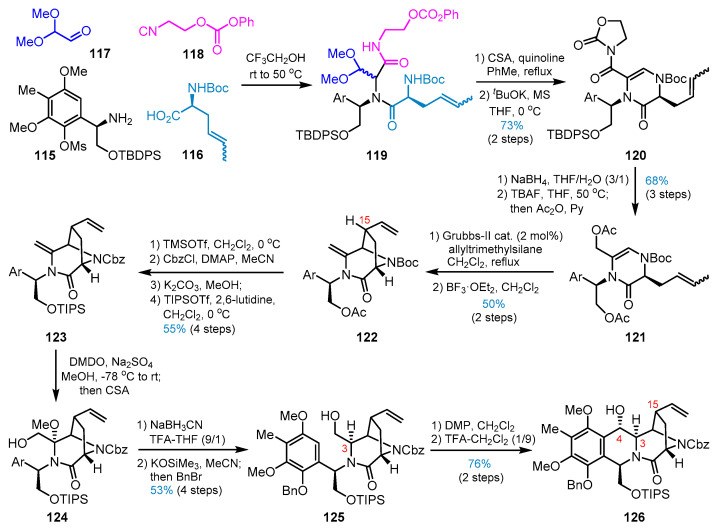
Fukuyama’s U-4CR approach toward the core of lemonomycin.

Early in 2004, the Fukuyama group developed an intriguing Ugi-4CR to assemble four readily prepared fragments for the rapid construction of the polycyclic core of lemonomycin in a highly convergent manner, although no subsequent synthesis was reported [54]. Amino alcohol **115**, amino acid **116**, glyoxyaldehyde acetal **117**, and isocyanide **118** were subjected to the Ugi reaction in CF_3_CH_2_OH to generate dipeptide **119**, followed by treatment with acid and cyclization under basic conditions to give *N*-acyloxazolidinone derivatives **120** in a good yield (Scheme 19). After the reduction and protection exchange, diacetate **121** was subjected to cross metathesis to introduce allyltrimethylsilane, which was elaborated to an intramolecular Hosomi–Sakurai reaction catalyzed by BF_3_•Et_2_O in an excellent yield, and complete stereochemical control at C15. To smoothly transform **122** into the desired alcohol **125**, they had to operate a four-step sequence of exchanging two protecting groups. The oxidation of enamide **123** by dimethyl dioxirane (DMDO) resulted in **124**, which upon treatment with TFA was reduced by NaBH_4_ to furnish the stereogenic center at C3 from a less hindered *exo*-face. Enhancement of the nucleophilicity of the aromatic ring by KOSiMe_3_ the promoted hydrolysis of the Ms group and benzylation in one pot. Alcohol **125** was oxidized by the Dess–Martin periodinane (DMP), and subsequent Friedel–Crafts cyclization yielded **126** with the requisite tetracyclic core of lemonomycin. Although they did not elaborate the current strategy to the final target, the key Hosomi–Sakurai cyclization was indeed successfully implemented into the later second-generation synthesis of lemonomycin (**1**) [31].

**Scheme 20:**
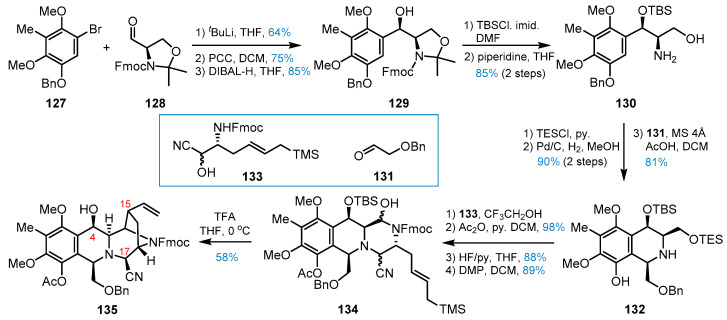
Mulzer’s cyclization approach toward the core of lemonomycin.

In 2008, Mulzer and coworkers completed a similar advanced intermediate by applying the intramolecular Hosomi–Sakurai reaction at different stages [55]. After a halogen exchange, the lithiation of arylbromide **127** reacted with aldehyde **128** to give a mixture of diastereomers of **129** (Scheme 20). An oxidation-reduction protocol secured a single diastereomer, which was then subjected to a protection–deprotection process to result in amino alcohol **130** in a good yield. After the removal of the benzyl group, the Pictet–Spengler reaction with aldehyde **131** proceeded under mild conditions to form the corresponding tetrahydroisoquinoline **132** in a highly selective manner. A Strecker-type condensation with cyanohydrin **133** was carried out to deliver hemiaminal **134** after oxidation of the primary alcohol at C16. Upon treatment with TFA, the reactive *N*-acyliminium ion-initiated Sakurai cyclization produced tetracyclic skeleton **135**. The epimerization of the cyano group at C17 was largely attributed to the less demanding equatorial position via an iminium ion intermediate. A phenomenon was commonly encountered in the syntheses of THIQ alkaloids [6,7].

**Scheme 21:**
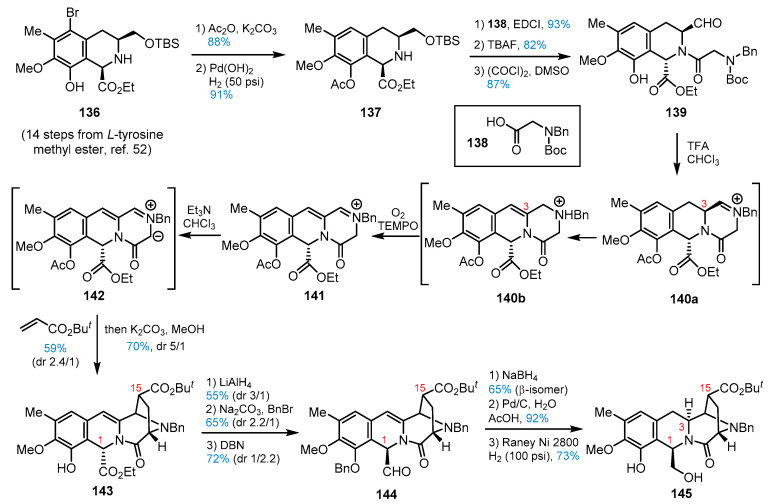
Williams’ intermolecular 1,3-dipolar cycloaddition approach [52].

The latest effort devoted to the synthesis of LMM was reported in 2013 by Williams and coworkers [56]. They devised a 1,3-dipolar cycloaddition of functionalized oxidopyrazinium at a late stage of synthesis (Scheme 21). The synthesis was commenced from tetrahydroisoqinoline **137**, which was derived from *L*-tyrosine methyl ester in 14 steps with a known protocol [57]. After acetylation and debromination, condensation with **139** and the subsequent transformation of the alcohol into aldehyde **140** resulted in a good overall yield. Treatment with TFA in CHCl_3_ generated an iminium salt intermediate that was readily oxidized in the presence of (2,2,6,6-tetramethylpiperidin-1-yl) oxidanyl (TEMPO) and oxygen, followed by the addition of triethylamine to result in oxidopyrazinium **143**. An immediate trap by *tert*-butyl acrylate smoothly afforded the requisite tetracyclic skeleton **144** with moderate facial selectivity (*dr* 2.4/1). The mixture was subjected to the following four steps: epimerization, partial reduction, benzylation, and isomerization at C1, and then the desired *β*-isomer at C1 was dominant. The reduction of aldehyde by NaBH_4_, debenzylation by Pd/C, and hydrogenation from the bottom face by Raney nickel under a high pressure of hydrogen eventually delivered the advanced intermediate **146**. The overall efficiency was largely detracted by the lengthy synthesis of aldehyde **140** and low diastereoselectivity of the intermolecular 1,3-dipolar cycloaddition. Therefore, a further transformation to aglycon **2** might not be facile due to the difficult accumulation of the key intermediate.

## 5. Conclusions

**Figure 3:**
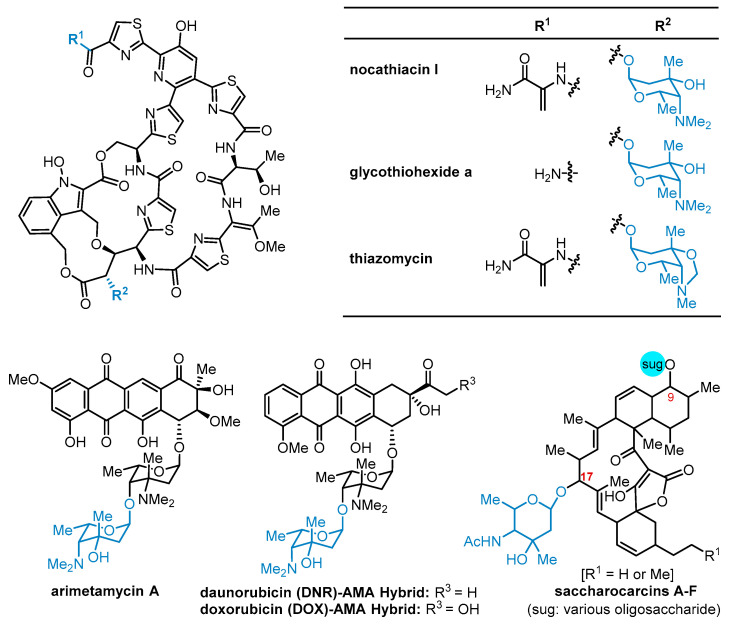
Antibiotics bearing 2,6-dideoxy-4-aminoglycoside (blue).

The carbohydrate motifs in various natural products play a pivotal role for the development of bioactive glycoconjugates and carrier prodrugs by attenuating toxicity and improving bioavailability [58]. The rare amino sugar lemonose was essentially implemented in complex metabolites, such as nocathiacin I [59], thiazamycin [60], glycothiohexide α [61], arimetamycin A [62], daunorubicin and doxorubicin hybrids [63], and various oligosaccharides in antitumor saccharocarcins A–F [64] (Figure 3). These antibiotics have been found to have potent antimicrobial activity against Gram-positive and Gram-negative pathogens, implicating the crucial impact of deoxyaminosugars.

Although various synthetic methods have been developed for the construction of deoxy aminosugars [65,66,67,68], the syntheses of lemonose and its derivatives were based mainly on *D*-threonine as the starting material, and the lengthy synthetic routes may render further modification [69]. Furthermore, since the latest synthetic efforts devoted to the synthesis of the LMM core in 2013 by the Williams group, there have been no reported efforts in the past decade. Efficient synthesis remains in high demand. Given the notorious difficulties in the chemical synthesis of complex structures in this specific family, enzymes are, therefore, an appealing alternative with excellent chemoselectivity, regioselectivity, and stereoselectivity [70]. With the development of gene recombination technology in recent years, metabolic engineering has emerged as a powerful tool for the accumulation of bulky materials and even complex targets [17,18,71]. The integration of biological and chemical methods encourages access to lemonomycin and its derivatives by gaining insights into the critical role of aminosugars and defining potent analogs for future antibiotic development to combat antibiotic-resistant infections.

## Data Availability

Not applicable.

## References

[1] O’Neill J. (2016). Tackling Drug-Resistant Infections Globally: Final Report and Recommendations.

[2] Butler M.S., Paterson D.L. (2020). Antibiotics in the clinical pipeline in October 2019. J. Antibiot..

[3] Whaley H.A., Patterson E.L., Dann M., Shay A.J., Porter J.N. (1964). Isolation and characterization of Lemonomycin, a new antibiotic. Antimicrob. Agents Chemother..

[4] Ashley E., Stoltz B. (2012). A Dipolar cycloaddition strategy for the synthesis of 3,8-diazabicyclo [3.2.1]octane tetrahydroisoquinoline antitumor antibiotics: The total synthesis of (-)-lemonomycin. Strateg. Tactics Org. Synth..

[5] He H.Y., Shen B., Carter G.T. (2000). Structural elucidation of lemonomycin, a potent antibiotic from *Streptomyces candidus*. Tetrahedron Lett..

[6] Scott J.D., Williams R.M. (2002). Chemistry and biology of the tetrahydroisoquinoline antitumor amtibiotics. Chem. Rev..

[7] Siengalewicz P., Rinner U., Mulzer j. (2008). Recent progress in the total synthesis of naphthyridinomycin and lemonomycin tetrahydroisoquinoline antitumor antibiotics (TAAs). Chem. Soc. Rev..

[8] Hill G.C., Wunz T.P., Remers W.A. (1988). Computer simulation of the binding of quinocarcin to DNA. Prediction of mode of action and absolute configuration. J. Comput. Aided Mol. Des..

[9] Sygusch J., Brisse F., Hanessian S., Kluepfel D. (1974). The molecular structure of naphthyridinomycin—A broad spectrum antibiotic. Tetrahedron Lett..

[10] Kluepfel D., Baker H.A., Piattoni G., Sehgal S.N., Sidorowicz A., Singh K., Vézina C. (1975). Naphthyridinomycin, a new broad-spectrum antibiotic. J. Antibiot..

[11] Zmijewski M., Mikolajczak M., Viswanatha V., Hruby V.J. (1982). Biosynthesis of the antitumor antibiotic naphthyridinomycin. J. Am. Chem. Soc..

[12] Zmijewski M., Palaniswamy V.A., Gould S.J. (1985). Naphthyridinomycin biosynthesis. The involvement of ornithine and the origin of the oxazolidine nitrogen. J. Chem. Soc. Chem. Commun..

[13] Palaniswamy V.A., Gould S.J. (1986). The incorporation of 3’-methyltyrosine and 5’-methyl DOPA into naphthyridinomycin. J. Am. Chem. Soc..

[14] Peng C., Pu J.Y., Song L.Q., Jian X.H., Tang M.C., Tang G.L. (2012). Hijacking a hydroxyethyl unit from a central metabolic ketose into a nonribosomal peptide assembly line. Proc. Natl. Acad. Sci. USA.

[15] Pu J.Y., Peng C., Tang M.C., Zhang Y., Guo J.P., Song L.Q., Hua Q., Tang G.L. (2013). Naphthyridinomycin biosynthesis revealing the use of leader peptide to guide nonribosomal peptide assembly. Org. Lett..

[16] Zhang Y., Wen W.-H., Pu J.-Y., Tang M.-C., Zhang L., Peng C., Xu Y., Tang G.-L. (2018). Extracellularly oxidative activation and inactivation of matured prodrug for cryptic self-resistance in naphthyridinomycin biosynthesis. Proc. Natl Acad. Sci. USA.

[17] Thibodeaux C.J., Melançon C.E., Liu H.W. (2008). Natural-product sugar biosynthesis and enzymatic glycodiversification. Angew. Chem. Int. Ed..

[18] Singh S., Phillips G.N., Thorson J.S. (2012). The structural biology of enzymes involved in natural product glycosylation. Nat. Prod. Rep..

[19] Hiratsuka T., Koketsu K., Minami A., Kaneko S., Yamazaki C., Watanabe K., Oguri H., Oikawa H. (2013). Core assembly mechanism of quinocarcin/SF-1739: Bimodular complex nonribosomal peptide synthetases for sequential Mannich-type reactions. Chem. Biol..

[20] Yang J., Song Y., Tang M., Li M., Deng J., Wong N., Ju J. (2021). Genome-directed discovery of tetrahydroisoquinolines from deep-sea derived Streptomyces niveus SCSIO 3406. J. Org. Chem..

[21] Wen W.-H., Zhang Y., Zhang Y.-Y., Yu Q., Jiang C.-C., Tang M.-C., Pu J.-Y., Wu L., Zhao Y.-L., Shi T. (2021). Reductive inactivation of the hemiaminal pharmacophore for resistance against tetrahydroisoquinoline antibiotics. Nat. Commun..

[22] Almabruk K.H., Dinh L.K., Philmus B. (2018). Self-resistance of natural product producers: Past, present, and future focusing on self-resistant protein variants. ACS Chem. Biol..

[23] Ashley E.R., Cruz E.G., Stoltz B.M. (2003). The total synthesis of (-)-lemonomycin. J. Am. Chem. Soc..

[24] Kiss M., Russell-Maynard J., Joule A. (1987). 1,3-Dipolar cycloadditions to oxidopyraziniums. Tetrahedron Lett..

[25] Allway P.A., Sutherland J.K., Joule J.A. (1990). A synthesis of (±)-7-methoxycarbonyl-2-(3-methoxyphenylmethylidene)-8-methyl-3,8-diazabicyclo[3.2.1] Octan-4-one (1b) using dipolar cycloaddition to a 3-oxidopyrazinium. Tetrahedron Lett..

[26] Yates N.D., Peters D.A., Allway P.A., Beddeoes R.L., Scopes D.I.C., Joule J.A. (1995). 1,3-Dipolar cycloadditions to oxidopyraziniums. Heterocycles.

[27] Reetz M.T., Schmitz A. (1999). Stereoselective Grignard-type reaction of chiral *N,N*-dibenzylamino ketones. Tetrahedron Lett..

[28] Reetz M.T. (1999). Synthesis and diastereoselective reactions of *N,N*-dibenzylamino aldehydes and related compounds. Chem. Rev..

[29] Pappo R., Allen D.S., Lemieux R.U., Johnson W.S. (1956). Osmium tetroxide-catalyzed periodate oxidation of olefinic bonds. J. Org. Chem..

[30] Thom C., Kocieński P. (1992). A practical method for *N*-acylation of bornane-2,10-sultam and 2-oxazolidinones. Synthesis.

[31] Yoshida A., Akaiwa M., Asakawa T., Hamashima Y., Yokoshima S., Fukuyama T., Kan T. (2012). Total synthesis of (-)-lemonomycin. Chem. Eur. J..

[32] Fukuyama T., Nunes J.J. (1988). Stereocontrolled total synthesis of (±)-quinocarcin. J. Am. Chem. Soc..

[33] Fukuyama T., Yang L., Ajeck K.L., Sachleben R.A. (1990). Total synthesis of (±)-saframycin A. J. Am. Chem. Soc..

[34] Mori K., Rikimaru K., Kan T., Fukuyama T. (2004). Synthetic studies on (+)-naphthyridinomycin:  Stereoselective synthesis of the tetracyclic core framework. Org. Lett..

[35] Majetich G., Lowery D., Khetani V., Song J., Hull K., Ringold C. (1991). Intramolecular additions of allylsilanes to conjugated dienones. Direct stereoselective syntheses of (±)-neolemnanyl acetate and (±)-neolemnane. J. Org. Chem..

[36] Gallina C., Liberatori A. (1974). Condensation of 1,4-diacetylpiperazine-2,5-dione with aldehydes. Tetrahedron.

[37] Ritter T., Stanek K., Larrosa I., Carreira E.M. (2004). Mild cleavage of aryl mesylates: Methanesulfonate as potent protecting group for phenols. Org. Lett..

[38] Mukaiyama T. (2004). Explorations into new reaction chemistry. Angew. Chem. Int. Ed..

[39] Magnus P., Matthews K.S. (2005). Synthesis of the tetrahydroisoquinoline alkaloid (±)-renieramycin G and a (±)-lemonomycinone analogue from a common intermediate. J. Am. Chem. Soc..

[40] Magnus P., Matthews K.S. (2012). A divergent strategy for synthesis of the tetrahydroisoquinoline alkaloids renieramycin G and a lemonomycin analog. Tetrahedron.

[41] Magnus P., Matthews K.S., Lynch V. (2003). New strategy for the synthesis of tetrahydroisoquinoline alkaloids. Org. Lett..

[42] Castro C.E., Havlin R., Honwad V.K., Malte A.M., Moje S.W. (1969). Copper(I) substitutions. Scope and mechanism of cuprous acetylide substitutions. J. Am. Chem. Soc..

[43] Kaufman T.S. (1997). Convenient one-pot synthesis of primary α-alkoxystannanes. Synlett.

[44] Yu C., Hu L. (2001). A facile synthesis of cyclic enecarbamates using Dess–Martin periodinane. Tetrahedron Lett..

[45] Pearlman W.M. (1967). Noble metal hydroxides on carbon nonpyrophoric dry catalysts. Tetrahedron Lett..

[46] Wu Y., Bernadat G., Masson G., Couturier C., Schlama T., Zhu J. (2009). Synthetic studies on (-)-lemonomycin: An efficient asymmetric synthesis of lemonomycinone amide. J. Org. Chem..

[47] Corey E.J., Xu F., Noe M.C. (1997). A rational approach to catalytic enantioselective enolate alkylation using a structurally rigidified and defined chiral quaternary ammonium salt under phase transfer conditions. J. Am. Chem. Soc..

[48] Lygo B., Wainwright P.G. (1997). A new class of asymmetric phase-transfer catalysts derived from Cinchona alkaloids–Application in the enantioselective synthesis of α-amino acids. Tetrahedron Lett..

[49] Lygo B., Andrews B.I. (2004). Asymmetric phase-transfer catalysis utilizing chiral quaternary ammonium salts: Asymmetric alkylation of glycine imines. Acc. Chem. Res..

[50] Kokotos G., Padrón J.M., Martín T., Gibbons W.A., Martin V.S. (1998). A general approach to the asymmetric synthesis of unsaturated lipidic α-amino acids. The first synthesis of α-aminoarachidonic acid. J. Org. Chem..

[51] Wu Y., Zhu J.P. (2008). Hafnium trifluoromethanesulfonate (hafnium triflate) as a highly efficient catalyst for chemoselective thioacetalization and transthioacetalization of carbonyl compounds. J. Org. Chem..

[52] Bernadat G., George N., Couturier C., Masson G., Schlama T., Zhu J. (2011). Asymmetric synthesis of 2,4,6-trideoxy-4-(dimethylamino)-3-*C*-methyl-*L*-lyxohexopyranose (lemonose). Synlett.

[53] Frigerio M., Santagostino M., Sputore S., Palmisano G. (1995). Oxidation of alcohols with o-iodoxybenzoic acid in DMSO: A new insight into an old hypervalent iodine reagent. J. Org. Chem..

[54] Rikimaru K., Mori K., Kan T., Fukuyama T. (2005). Synthetic studies on (–)-lemonomycin: Stereocontrolled construction of the 3,8-diazabicyclo[3.2.1] skeleton. Chem. Commun..

[55] Siengalewicz P., Brecker L., Mulzer J. (2008). Stereocontrolled synthesis of the tetracyclic core framework of (-)-lemonomycin. Synlett.

[56] Jiménez-Somarribas A., Williams R.M. (2013). Synthetic studies on lemonomycin: Construction of the tetracyclic core. Tetrahedron.

[57] Liao X.W., Liu W., Dong W.F., Guan B.H., Chen S.Z., Liu Z.Z. (2009). Total synthesis of (−)-renieramycin G from *l*-tyrosine. Tetrahedron.

[58] Kren V., Rezanka T. (2008). Sweet antibiotics-the role of glycosidic residues in antibiotic and antitumor activity and their randomization. FEMS Microbiol. Rev..

[59] Constantine K.L., Mueller L., Huang S., Abid S., Lam K.S., Li W., Leet J.E. (2002). Conformation and absolute configuration of nocathiacin I determined by NMR spectroscopy and chiral capillary electrophoresis. J. Am. Chem. Soc..

[60] Zhang C., Herath K., Jayasuriya H., Ondeyka J.G., Zink D.L., Occi J., Birdsall G., Venugopal J., Ushio M., Burgess B. (2009). Thiazomycins, thiazolyl peptide antibiotics from *Amycolatopsis fastidiosa*. J. Nat. Prod..

[61] Northcote P.T., Siegel M., Borders D.B., Lee M.D. (1994). Glycothiohexide α, a novel antibiotic produced by “Sebekia” sp., LL-14E605. III. Structural elucidation. J. Antibiot..

[62] Kang H.S., Brady S.F. (2013). Arimetamycin A: Improving clinically relevant families of natural products through sequence-guided screening of soil metagenomes. Angew. Chem. Int. Ed..

[63] Huseman E.D., Byl J.A.W., Chapp S.M., Schley N.D., Osheroff N., Townsend S.D. (2021). Synthesis and cytotoxic evaluation of arimetamycin A and its daunorubicin and doxorubicin hybrids. ACS Cent. Sci..

[64] Hegde V.R., Patel M.G., Das P.R., Pramanik B., Puar M.S. (1997). A family of novel macrocyclic lactones, the saccharocarcins produced by *Saccharothrix aerocolonigenes subsp. antihiotica*. II. Physico-chemical properties and structure determination. J. Antibiot..

[65] Pfrengle F.H.-U. (2010). Reissig, Amino sugars and their mimetics via 1,2-oxazines. Chem. Soc. Rev..

[66] Ding F., Cai S., William R., Liu X.-W. (2013). Pathways leading to 3-amino- and 3-nitro-2,3-dideoxy sugars: Strategies and synthesis. RSC Adv..

[67] Skarbek K., Milewska M.J. (2016). Biosynthetic and synthetic access to amino sugars. Carbohydr. Res..

[68] Behera A., Kulkarni S.S. (2018). Chemical synthesis of rare, deoxy-amino sugars containing bacterial glycoconjugates as potential vaccine candidates. Molecules.

[69] Huseman E.D., Townsend S.D. (2021). De novo synthesis of an *L*-lemonose thioglycoside donor from *D*-Threonine. Tetrahedron Lett..

[70] Gloster T.M. (2020). Exploitation of carbohydrate processing enzymes in biocatalysis. Curr. Opin. Chem. Biol..

[71] Lee S.Y., Kim H.U., Chae T.U., Cho J.S., Kim J.W., Shin J.H., Kim D.I., Ko Y.-S., Jang W.D., Jang Y.-S. (2019). A comprehensive metabolic map for production of bio-based chemicals. Nat. Catal..

